# Standardized Methods for Evaluating Physical and Eating Behaviors: The WEALTH Cross-Sectional Study Protocol

**DOI:** 10.2196/70186

**Published:** 2026-03-06

**Authors:** Grainne Hayes, Christoph Buck, Greet Cardon, Richard Cimler, Steriani Elavsky, Leopold Fezeu K, Janas M Harrington, Jitka Kühnová, Jean-Michel Oppert, Luis Sigcha, Pepijn Van de Ven, Tomas Vetrovsky, Catherine B Woods, Antje Hebestreit, Alan E Donnelly

**Affiliations:** 1 Department of Physical Education and Sport Sciences, Health Research Institute, Physical Activity for Health Research Centre, University of Limerick, Limerick, Ireland, 353 860806576; 2 Leibniz Institute for Prevention Research and Epidemiology - BIPS, Bremen, Germany; 3 Department of Movement and Sports Sciences, Ghent University, Ghent, Belgium; 4 Faculty of Science, University of Hradec Králové, Hradec Králové, Czech Republic; 5 Department of Human Movement Studies, University of Ostrava, Ostrava, Czech Republic; 6 Université Sorbonne Paris Nord et Université Paris Cité, INSERM U1153, INRAE U1125, CNAM, Centre de Recherche en Epidémiologie et Statistiques (CRESS), Équipe de Recherche en Épidémiologie Nutritionnelle (EREN), Bobigny, France; 7 Centre for Health and Diet Research, School of Public Health, University College Cork, Cork, Ireland; 8 Department of Nutrition, Pitie-Salpetriere Hospital (AP-HP), Sorbonne University, Paris, France; 9 Health Research Institute, University of Limerick, Limerick, Ireland

**Keywords:** physical behaviors, eating behaviors, ecological momentary assessment, machine learning, wearables

## Abstract

**Background:**

The accurate measurement of physical behaviors (PBs) and eating behaviors (EBs) is critical for designing, monitoring, and implementing public health guidelines and intervention strategies. The objective of the Wearable Sensor Assessment of Physical and Eating Behaviours (WEALTH) project was to develop standardized methods to identify daily PBs and EBs from wearable research- and consumer-grade sensors and evaluate the interaction and contexts of these behaviors.

**Objective:**

The aim of this paper is to describe the study design and methods and report on the descriptive characteristics of the participants.

**Methods:**

Within the framework of the WEALTH project, a cross-sectional study (spring 2023 to spring 2024) was completed in 5 European research centers in the Czech Republic, France, Germany, and Ireland. In each center, participants attended a research lab, completed an online questionnaire, and provided measures of anthropometry and handgrip strength. The participants were then fitted with 2 research-grade and 2 consumer-grade devices and participated in a standardized semistructured lab-based activity protocol. The latter was specifically designed to collect labeled data that simulated common PBs and EBs typical for a daily routine. Participants were then followed during a 9-day free-living data collection period, which combined the assessment of PB and EB via wearable devices and time-based, event-based, and self-initiated ecological momentary assessments (EMAs). The EMA surveys were complemented by three 24-hour dietary recalls, using validated web-based programs. Upon the completion of the survey protocol, participants completed a questionnaire that assessed the feasibility of the procedures.

**Results:**

The final sample includes 627 participants, of whom 44% (n=275) were male. The mean age was 32.7 (SD 13.3) years, and the mean body mass index was 24.5 (SD 4.0) kg/m². The WEALTH study data will be used to develop machine learning (ML) models for classifying daily activities from wrist and hip-worn accelerometer data, evaluate EMA methods for studying interactions between PB and EB, and evaluate the feasibility and compliance of the methods. Data processing and ML model development are currently underway, with primary results expected to be published in 2026.

**Conclusions:**

The output of the WEALTH project will be provided via a repository and a comprised toolbox of publicly available labeled data, ML models for behavior classification from accelerometer data, and a methodology to simultaneously capture EB and PB, thereby producing an integrated data collection system to support future research.

## Introduction

Engaging in healthy physical behaviors (PBs), including regular physical activity (PA), low sedentary behavior, and optimal sleep routines, as well as in healthy eating behaviors (EBs), is essential for reducing the risk of noncommunicable diseases [1,2]. The accurate measurement of PBs is critical for designing, monitoring, and implementing public health guidelines and interventions. However, most existing assessment methodologies rely on self-report measures, which are susceptible to recall bias and social desirability bias and often demonstrate weak validity and reliability [3]. These limitations restrict the ability to examine how PBs interact with eating patterns and behaviors, collectively referred to here as EBs [4].

Device-based measures have improved the objectivity of PB assessment in epidemiological research [5]. Yet, comparisons across studies remain challenging due to differences in research-grade devices, nonstandardized intensity cut-points, and inconsistent validation analysis techniques [6]. Consumer-grade wearables are increasingly accessible, but their validity varies widely across device models and manufacturers [7-9], and many do not meet the standards required for academic research [9,10]. While conventional approaches to processing accelerometer data have relied on heuristic methodologies, machine learning (ML) techniques now enable more precise estimations [11,12]. These advancements allow for the improved classification of PB and energy expenditure from raw accelerometer data [13], compared with cut-point–based approaches [14]. However, standardized protocols for implementing ML in this context (PB) are still lacking.

Similar methodological inconsistencies affect EB measurement. Emerging digital and sensing technologies enable the objective and passive measurement of EBs; for example, wearable devices, such as smart watches, are equipped with accelerometers and gyroscopes that can track hand-to-mouth movements and hand gestures, providing insights into eating frequency, speed, and duration, as well as eating-related activities [15-17]. These technologies still encounter limitations when used in free-living environments [15,18]; for example, the diversity of available devices and sensors contributes to inconsistencies in data collection and analysis; thus, the absence of standardized measurement methodologies hinders cross-study comparisons and data interpretation. As a result, integrating PB and EB data in a meaningful way remains challenging.

Standardized measurement approaches (ie, standardized accelerometers, instruments, protocols, processing procedures) are therefore essential for producing comparable, high-quality PB and EB data and for enabling the examination of how these behaviors co-occur in daily life. Such standardization ensures that PB and EB data can be meaningfully integrated and compared. Despite the advantages of accelerometers for capturing movement-related aspects of PBs, these devices cannot provide information on the contextual factors that shape either EB or PB. Ecological momentary assessment (EMA) can address this limitation by capturing real-time self-reported data on behaviors and their surrounding contexts. By leveraging mobile technologies, EMA offers insight into the environmental, social, and psychological circumstances in which behaviors occur, thereby complementing accelerometry-based measures and strengthening multimethod approaches to PB and EB assessment [19]. At the same time, ML techniques have the potential to enable the use of sensor data from wrist-worn devices to detect and classify EB, such as meal onset or eating speed [18], but harmonized and validated models applicable across devices are still lacking.

Together, EMA and ML can complement conventional measures and contribute to more precise and integrated assessments of PBs and EBs. A web-based data processing framework incorporating ML models trained and validated for the processing and analysis of wearable device data that works seamlessly both for existing research-grade and commercial devices is feasible and warranted. To address the identified gaps, the aim of the “Wearable Sensor Assessment of Physical and Eating Behaviours” (WEALTH) project was to develop standardized methods for classifying daily PBs and EBs from accelerometry or sensor devices using EMA and ML methods and to investigate the interrelationship of both behaviors. To achieve this, the objectives of WEALTH were to (1) establish the feasibility of integrating accelerometer-derived PB data with EMA-derived EB data in free-living environments; (2) generate high-quality, standardized datasets using harmonized protocols; and (3) develop and validate ML models capable of classifying PBs. Secondary objectives included the creation of a web-based data processing infrastructure to support ML implementation, cross-device compatibility, and open-source data workflows and additional feasibility outcome measures. This paper describes the design and methods of the WEALTH project, providing insights into the available data and the quality measures to derive this unique dataset.

## Methods

### Study Design

Initiated in 2022, the WEALTH project was funded by the EU Joint Programming Initiative Healthy Diet Healthy Life [20] under the call on STAMIFY (standardised measurement, monitoring and/or biomarkers to study food intake, physical activity and health).

In the framework of the WEALTH project, a multicountry mixed methods cross-sectional survey was conducted, which focused on enrolling and monitoring PBs and EBs of adult participants from 5 research centers across Europe, in the Czech Republic, France, Germany, and Ireland. Participants were invited to participate between March 2023 and March 2024 via stratified and convenience sampling methods through letters, emails, telephone calls, posters, flyers, and social media. France was the exception, recruiting their participants from the NutriNet-Santé study. With a target sample size of 600 participants (150 participants: 50% (n=75) female, aged 18‐64 years per country), enrollment was distributed across the centers meeting the project’s eligibility criteria (see below). A small cash reward incentive of €30-€40 (US $35-$47) per participant was given in the Czech Republic, Germany, and Ireland to increase response rates, participant compliance, and ensure the return of all devices. Participants who dropped out during the study or provided insufficient data were eliminated. Participants who dropped out before the center total was reached were replaced. A total of 627 adults completed the study (Table 1).

**Table 1.** Study population baseline characteristics.

		Total			Male			Female		
		Participants, n	Mean (SD)	IQR	Participants, n (%)	Mean (SD)	IQR	Participants, n (%)	Mean (SD)	IQR
Total										
Height (m)		627	1.7 (0.1)	1.6-1.8	275 (43.9)	1.8 (0.1)	1.7-1.8	352 (56.1)	1.7 (0.1)	1.6-1.7
Weight (kg)		627	72.1 (14.3)	61.2-80.8	275 (43.9)	80.8 (12.7)	72.9-88.0	352 (56.1)	65.3 (11.6)	56.9-70.8
BMI (kg/m^2^)		627	24.5 (4.0)	21.6-26.6	275 (43.9)	25.1 (3.7)	22.8-26.9	352 (56.1)	24.0 (4.1)	21.1-26.1
Age (y)		627	32.7 (13.3)	24.0-40.0	275 (43.9)	33.2 (13.0)	24.0-41.0	352 (56.1)	32.3 (13.5)	24.0-40.0
Waist circumference (cm)		626	83.7 (11.9)	74.2-91.0	274 (43.8)	89.5 (10.9)	81.3-95.0	352 (56.1)	79.1 (10.6)	71.2-86.0
Max handgrip strength (kg)		627	35.1 (10.7)	26.9-43.2	275 (43.9)	44.5 (7.9)	39.1-50.0	352 (56.1)	27.7 (5.6)	23.9-31.5
Czech Republic										
Height (m)		162	1.7 (0.1)	1.6-1.8	60 (37.0)	1.8 (0.1)	1.8-1.9	102 (63.0)	1.7 (0.1)	1.6-1.7
Weight (kg)		162	74.1 (15.6)	61.2-83.9	60 (37.0)	83.4 (14.3)	74.6-88.9	102 (63.0)	68.5 (13.7)	58.3-76.4
BMI (kg/m^2^)		162	24.9 (4.4)	21.7-26.9	60 (37.0)	25.3 (3.7)	23.3-26.6	102 (63.0)	24.7 (4.7)	21.3-27.4
Age (y)		162	37.4 (11.4)	29.0-46.0	60 (37.0)	36.7 (9.9)	29.0-44.0	102 (63.0)	37.8 (12.2)	29.0-47.0
Waist circumference (cm)		161	84.7 (11.3)	74.5-92.0	59 (36.6)	89.6 (10.4)	81.0-95.5	102 (63.0)	81.9 (10.9)	72.5-89.5
Max handgrip strength (kg)		162	34.7 (10.9)	27.7-42.0	60 (37.0)	45.2 (8.8)	38.6-50.5	102 (63.0)	28.5 (6.4)	25.2-32.3
France										
Height (m)		159	1.7 (0.1)	1.6-1.8	68 (42.8)	1.8 (0.1)	1.7-1.8	91 (57.2)	1.6 (0.1)	1.6-1.7
Weight (kg)		159	72.5 (14.6)	61.3-82.4	68 (42.8)	81.5 (13.1)	73.8-90.4	91 (57.2)	65.8 (11.8)	56.6-71.5
BMI (kg/m^2^)		159	25.2 (4.4)	22.0-27.5	68 (42.8)	25.9 (4.3)	22.4-28.5	91 (57.2)	24.6 (4.4)	21.2-26.8
Age (y)		159	36.7 (17.6)	20.0-57.0	68 (42.8)	39.3 (17.7)	21.0-57.5	91 (57.2)	34.7 (17.3)	20.0-52.0
Waist circumference (cm)		159	85.7 (14.2)	74.5-94.0	68 (42.8)	93.4 (13.0)	84.5-104.4	91 (57.2)	80.0 (12.2)	71.4-86.0
Max handgrip strength (kg)		159	33.8 (9.9)	25.4-42.2	68 (42.8)	43.0 (7.5)	39.5-47.5	91 (57.2)	27.0 (4.6)	23.5-30.8
Germany										
Height (m)		156	1.7 (0.1)	1.6-1.8	72 (46.2)	1.8 (0.1)	1.7-1.8	84 (53.8)	1.6 (0.1)	1.6-1.7
Weight (kg)		156	68.7 (12.5)	60.0-76.1	72 (46.2)	76.1 (11.9)	68.5-84.0	84 (53.8)	62.3 (9.1)	55.3-67.3
BMI (kg/m^2^)		156	23.7 (3.3)	21.3-25.6	72 (46.2)	24.1 (3.3)	21.6-26.0	84 (53.8)	23.4 (3.2)	21.2-25.4
Age (y)		156	24.3 (2.0)	24.0-25.0	72 (46.2)	24.5 (0.5)	24.0-25.0	84 (53.8)	24.1 (2.7)	24.0-25.0
Waist circumference (cm)		156	82.6 (10.6)	74.4-89.2	72 (46.2)	87.9 (10.4)	80.7-93.5	84 (53.8)	78.1 (8.4)	71.2-85.2
Max handgrip strength (kg)		156	32.6 (9.9)	24.5-39.7	72 (46.2)	41.4 (7.0)	37.4-46.5	84 (53.8)	25.1 (4.1)	22.1-27.8
Ireland										
Height (m)		150	1.7 (0.1)	1.7-1.8	75 (50)	1.8 (0.1)	1.8-1.9	75 (50)	1.7 (0.1)	1.6-1.7
Weight (kg)		150	73.1 (13.9)	62.0-81.5	75 (50)	82.6 (10.4)	76.5-88.1	75 (50)	63.7 (10.0)	57.0-67.7
Body Mass Index (kg/m^2^)		150	24.2 (3.5)	21.5-26.0	75 (50)	25.4 (3.2)	23.2-27.1	75 (50)	23.0 (3.5)	20.3-25.0
Age (y)		150	32.2 (12.3)	22.0-40.0	75 (50)	33.1 (12.1)	23.0-41.0	75 (50)	31.3 (12.6)	22.0-39.0
Waist circumference (cm)		150	81.4 (10.8)	72.5-88.0	75 (50)	87.5 (8.7)	81.0-92.0	75 (50)	75.4 (9.3)	67.5-82.0
Max handgrip strength (kg)		150	39.5 (10.9)	31.2-48.7	75 (50)	48.4 (6.7)	44.0-52.5	75 (50)	30.5 (5.6)	27.5-33.5

### Eligibility Criteria

To be deemed eligible for inclusion, participants were required to be aged between 18 and 64 years. To maintain protocol homogeneity and ensure the relevance of the data processing methods applied, individuals with overt chronic diseases, physical impairments, and those engaged in shift work were excluded from the study. These exclusion criteria were implemented to uphold the integrity of the prescribed semistructured activity protocol and to prevent potential disturbances in the free-living period arising from participants’ unique circumstances and, consequently, enhance the fidelity of the ensuing analytical outcomes.

### Study Procedures and Data Collection: Overview of Study Protocol

**Figure 1.**
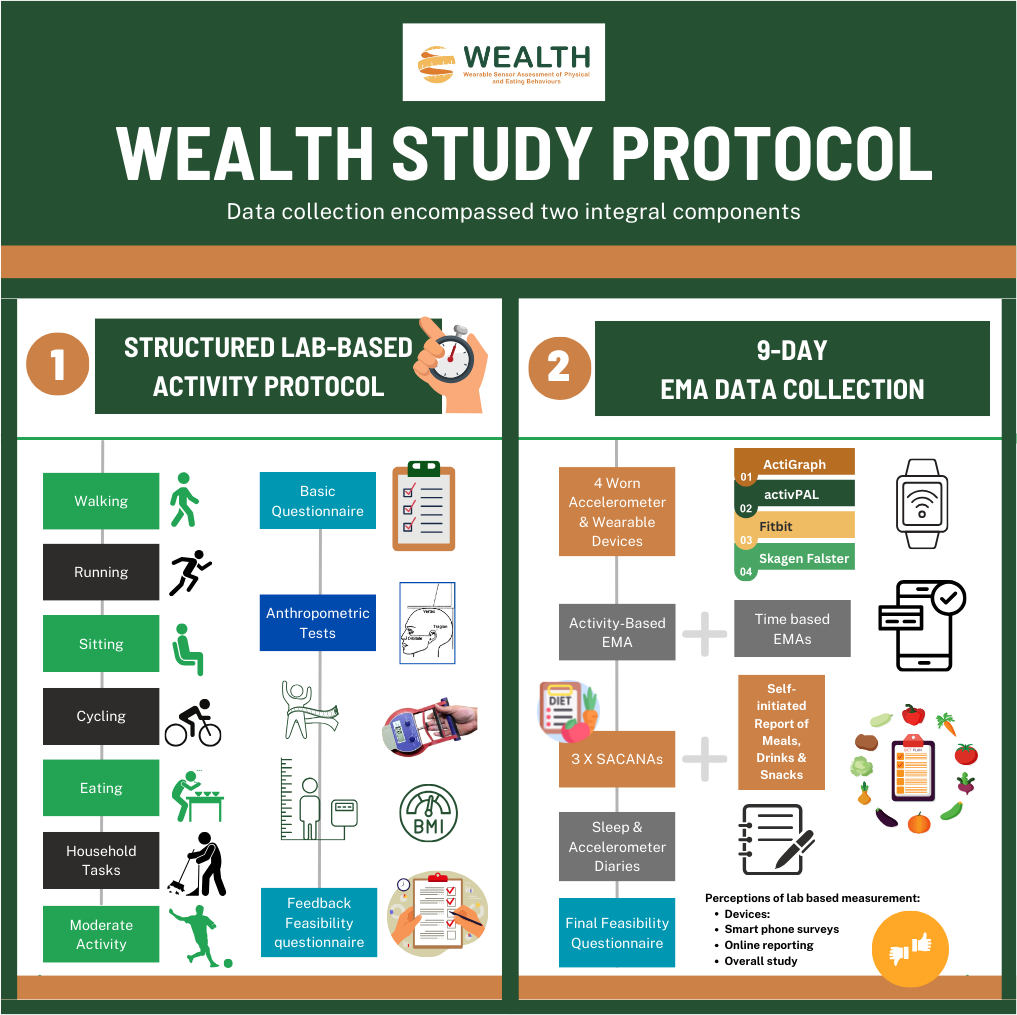
WEALTH (Wearable Sensor Assessment of Physical and Eating Behaviours) study protocol. EMA: ecological momentary assessment.

The WEALTH data collection encompassed 2 integral components (Figure 1). The first component involved a standardized semistructured lab–based activity protocol, specifically designed to collect labeled data that simulated common PBs and EBs. The second component extended the data collection beyond the laboratory setting. It incorporated a 9-day EMA data collection combined with worn accelerometer and wearable devices, thereby enabling the continuous data collection of individuals’ behaviors during free-living. To record the participants’ experience of the study, an end-of-study feasibility instrument was completed when the participants returned their devices. To uphold methodological rigor and ensure conformity across all stages of the study, a WEALTH standard operating procedures manual was developed to define the WEALTH research group’s study protocol consisting of different modules (wearables, anthropometric measurements, dietary assessment, and general questionnaire), field access with interview guidelines, order of measurements, measurement procedures, equipment requirements, prerequisites, and data entry. The manual served as a comprehensive reference tool, offering researchers clear guidelines to enhance the precision and effectiveness of the data collection processes across the various study locations.

### Measurement of Baseline Information and Anthropometric Characteristics

#### Measurement of Baseline Information

Upon participant recruitment and prior to participating in the laboratory testing session, participants were required to provide online written consent, download the Fitbit app and a custom-built mobile app HealthReact for delivering the EMA surveys, and complete a Physical Activity Readiness Questionnaire [21].

Participants attended the center wearing light gym clothing and running shoes. Upon arrival, they were provided with a verbal description of the semistructured laboratory and free-living components of the study. In Ireland, participants then completed an online baseline WEALTH questionnaire, while researchers verified the installation of the HealthReact and Fitbit apps. In the Czech Republic and France, participants filled in the online baseline WEALTH questionnaire through a secured link prior to participating in the laboratory session. In Germany, participants were provided with a link to the online baseline WEALTH questionnaire and were asked to fill it out in the upcoming week. The general WEALTH questionnaire comprised several instruments assessing important co-variates, such as participant sociodemographics and lifestyle behaviors, such as smoking, alcohol drinking, sleep duration, PA, and food consumption. It also assessed the general health status, including the Short Form 36 Health Survey Questionnaire instrument [22], to assess health-related quality of life. All questionnaires were provided in the language of the respective country (translated and back translated to control for translation errors or using established translated versions of the tools).

#### Measurement of Anthropometric Characteristics

Anthropometric measurements of the participants were measured without shoes and in light clothing. Height was measured to the nearest 0.25 cm, using a portable stadiometer (Seca Ltd), and body mass was measured to the nearest 0.1 kg using a portable electronic scale (Seca Ltd). BMI was recorded using the standard formula (kg/m^2^). Waist circumference was recorded to the nearest 0.1 cm with an adjustable anthropometric inelastic tape (Seca Ltd). The measurement was taken at the level of the narrowest point of the abdomen at the end of normal exhalation as observed from the anterior aspect. Alternatively, if no one point was evident, waist circumference was assessed at the midpoint of the lowest rib and iliac crest at the end of normal exhalation. The measurement of the waist circumference was completed twice.

Handgrip strength was measured with the dominant and nondominant hand using a digital hand dynamometer with adjustable grip (Takei Scientiﬁc Instruments Co). Participants were instructed to self-adjust the dynamometer so that it fitted comfortably to their hand size to obtain their best performance [23]. Prior to the measurement, participants were taken through a familiarization trial for each hand so that they could get acquainted with the instrument and procedures and choose the best adjustment [24]. Participants were then instructed to grip the dynamometer with maximum strength in response to a voice command [25]. For the measurements, participants stood holding the dynamometer by their side with both arms fully extended, avoiding contact with any other body part [26]. Two trials were performed on each side, alternately, with a rest period of at least 1 minute between trials of the same hand [27].

#### Measurement of PBs and EBs

Participants were fitted with 2 research-grade (ActiGraph wGT3X-BT, activPAL 3 micro) and 2 consumer-grade (Fitbit Charge 5, LifeQ enabled smartwatch) devices [28]. The Fitbit (Fitbit) and a LifeQ-enabled smartwatch (Skagen Designs Generation 6) were worn on the participants’ nondominant and dominant wrist, respectively. The smartwatch was supported by LifeQ to enable the storage and download of tri-axial accelerometer data and heart rate from green photoplethysmography. The Fitbit device was synchronized with the Health React app on either the participant’s or the study smartphone. The number of steps and heart rate data from Fitbit were collected on the Health React server. The ActiGraph wGT3X-BT was attached to the right iliac crest using an elastic band, and the activPAL was attached to their right thigh as per manufacturer guidelines [28]. The characteristics and specifications of each activity monitor are described in Table 2. The activPAL device has demonstrated excellent reliability and validity in measuring sedentary behavior and PA patterns in free-living conditions [28]. Similarly, the ActiGraph device has shown robust reliability and validity in assessing PA levels across various populations and settings [29,30]. Favorable reliability and validity metrics have been reported for the Fitbit Charge 5, particularly in tracking steps, heart rate, and sleep patterns [31]. While the LifeQ-enabled smartwatch shows promising reliability and validity in monitoring PA and providing personalized feedback, further research is needed to establish its performance fully [32]. To address the potential risk of timestamp misalignment across the 4 wearable devices, all devices were synchronized to a single study laptop or to a server time prior to deployment, ensuring consistent timing across devices. Continuous data collection protocols will incorporate periodic checks and automatic synchronization routines (where supported by the device manufacturer) to maintain temporal alignment throughout the monitoring period. Any residual discrepancies in timestamps will be handled during the data curation process prior to the use in ML pipelines.

**Table 2.** Device specifications.

Specification	activPAL 3 micro	ActiGraph wGT3X-BT	LifeQ-enabled smartwatch	Fitbit Charge 5
Size (mm)	23.5×43×5	33×46×15	32.5 active area diameter	36.8×23×11
Mass (g)	10	19	100	29
Placement	Midpoint of anterior right thigh	Right iliac crest	Dominant wrist	Nondominant wrist
Application	Waterproofed with nitrile sleeve and tegaderm	Elastic Belt	Wrist strap	Wrist strap
Internal sensors	Tri-axial accelerometer	Tri-axial Accelerometer	Uni-axial accelerometer, heart rate monitor	Tri-axial accelerometer, heart rate monitor
Sample rate (Hz)	20‐80	30‐100	25	100
Accelerometer amplitude range (±g)	6	2‐8	Not reported	Not reported
Connectivity (Wifi or Bluetooth or USB)	USB	USB and Bluetooth	Wifi, USB, and Bluetooth	USB and Bluetooth
Battery life	7‐10 days	7‐14 days	24‐36 hours	7 days

In the German study center, a subsample of participants was fitted with a portable metabolic unit (MetaMax 3b, CORTEX Biophysik, Leipzig) for the duration of the standardized activity protocol. Oxygen uptake (VO_2_ mL/kg/min), carbon dioxide production (VCO_2_, mL/kg/min), and heart rate were measured breath-by-breath. The data were internally stored during the standardized activity protocol and uploaded to the computer running the dedicated software (MetaSoft, Cortex Biophysik, Leipzig) afterward.

For collecting labeled data, the participants then followed a 2-hour standardized semistructured activity protocol to record PBs and EBs, designed to emulate activities of daily living. The participants performed a maximum of 11 common activities (Table 3) across 4 intensity categories (sedentary, light, moderate, and vigorous). Sedentary and light-intensity activities were performed for a duration of 3‐5 minutes, while moderate- and vigorous-intensity activities were performed for a duration of 4‐10 minutes. The eating a meal task was performed over a duration of 10 minutes.

**Table 3.** Standardized semistructured activity protocol.

Time (min)	Activity intensity	Description
10	Sedentary	Participants sit and fill in cross-word puzzles (5 min) and watch the news (5 min).
6	Standing and snacking	Passive standing: relaxed (3 min). Stand and snack while watching the TV^a^ (3 min).
5	Light activity	Slow walking: individual speed, not controlled. Include 2 random stops for about 20 seconds (5 min).
5	Moderate activity	Brisk walking: individual speed, not controlled (5 min).
4	Vigorous activity	Jogging or running: individual speed, not controlled (4 min).
3	Transition	Short rest: provide water especially to participants with spirometry masks. Allow participants to sit, check phones, and so forth. Explain games and the next task.
8	Moderate activity	Let participants choose active games and switch in between (eg, 1 vs 1 for each game). Participants do not need to attend all possible games but rather games that they are able or experienced to play (8 min). Football playing soccer back and forth Badminton Table tennis Basketball (no competition rather playing to the basket)
4	Transition	Get the bikes and give directions (4 min)
5	Moderate activity	Cycling: cycle round outside of the building on a real bike or stationary bike. Ensure that participants have previously cycled and are comfortable in doing so. All participants are required to wear a helmet throughout this task. Include at least 2 stops for about 20 seconds (5 min).
5	Rest	Short-seated: rest particularly for the participant wearing spirometry—ask participants if they want to keep on the masks—provide water. Explain household tasks for next tasks (5 min).
10	Light activity	Household tasks: let participants switch between tasks, for example, participant A starts hoovering, participant B is filling the dishwasher, then participant A is emptying the dishwasher and B is hoovering. Participants do not need to execute all provided tasks (10 min). Hoovering Sweeping or mopping (without water) Emptying or filling the dishwasher Chopping vegetables Spread butter on toast, prepare sandwiches for task 9 (could be seated at the table).
10	Eating a meal (with drinks)	Eat and drink while sitting provided food and drinks. Sandwiches, vegetables or fruits, yogurt (provide different options: vegan, with milk, plain, or with flavor). Proposed sequence: Minute 1: drink out of a cup (cold beverage) Minute 2‐3: Use knife or fork Minute 4‐5: Use spoon (cereal or yogurt) Minute 6‐7: Eat with hand From minute 7: let participants eat as preferred Activity session ends after eating a meal task.

a TV: television.

### 9-Day Free-Living Data Collection

#### Time-Based, Event-Based, and Self-Initiated Ecological Momentary Assessments

Following the standardized activity protocol, participants were required to continue to wear the 4 devices during a 9-day free-living data collection. During this time, participants received time-based and event-based EMA prompts to answer short questionnaire surveys that appeared on their smartphones. In addition, they were instructed to self-initiate surveys on specific occasions when they were eating, snacking, or drinking (except water).

The EMA data were collected using the HealthReact platform developed at the University of Hradec Kralove, Czechia (a license to use HealthReact is held by a company owned by Richard Cimler, a member of the WEALTH consortium). HealthReact is a software suite comprising a server-side component and a mobile app for iOS and Android smartphones [33]. The HealthReact server can collect data from a diverse range of sensors, including wearable devices, such as Fitbit, and it can evaluate these data in real time to automatically trigger event-based EMA surveys based on predefined rules. These rules can be customized by researchers through a user-friendly web interface. The EMA surveys are then pushed to and displayed on the HealthReact app on participants’ smartphones. Additionally, HealthReact supports traditional time–based surveys, which are triggered at random times within a specified time frame. Finally, HealthReact also enables the possibility of self-initiated surveys. For the WEALTH study, the Fitbit Charge 5 was chosen as a suitable wearable sensor for triggering the event-based surveys within HealthReact. While HealthReact is not the only platform that enables accelerometry-based triggers, most commonly used EMA platforms do not support this feature, which may limit comparability with other studies.

For the 9-day EMA protocol, the data collection was standardized, with the day when participants attended the lab (day -1) and the first full day (day 0) considered as “lead-in” days, followed by 7 full days of the monitoring period (ie, day 1 -to day 7). Participants without a compatible smartphone or without a mobile data plan were provided with a project smartphone, which included a continuous mobile internet connection. The EMA protocol consisted of a combination of time-based, event-based, and self-initiated surveys. There were 7 time-based surveys (08:00-09:45 morning survey, 10:15-11:45, 12:15-13:45, 14:15-15:45, 16:15-17:45, 18:15-19:45 daily surveys, 20:15-21:45 evening survey) triggered randomly in the predefined time windows. These windows were designed with a time gap to ensure the surveys were 30 minutes apart. In addition to time-based surveys, the event-based surveys were triggered by data collected by the Fitbit Charge 5 that was worn on the participants’ wrists. The Fitbit device provided accurate minute-level number of steps [34] and several heart rate recordings per minute. These data were collected continuously and required continuous internet connection for regular synchronization with the Fitbit server, which occurs automatically approximately every 15 minutes or when the participant manually syncs the app. A sedentary survey was triggered after 20 consecutive minutes of 0 steps with concurrent detection of heart rate to distinguish between sedentary behavior and nonwear. A maximum of 2 sedentary surveys were administered within the 8:00-14:00 time window and the 14:00-20:00 time window (ie, a maximum of 4 surveys per day), and there was a minimum gap of 90 minutes between 2 sedentary surveys. A walking survey was triggered after 5 consecutive minutes with ≥60<140 steps/minute, with a maximum of 3 walking surveys per day. A running survey was triggered after 5 consecutive minutes with ≥140 steps/minute, with a maximum of 3 running surveys per day. It should be noted that because the triggers were step-based, the event-based EMA surveys did not capture nonambulatory forms of PA (eg, cycling, resistance training, yoga). These activities could still be reported as “exercise” via time-based or self-initiated surveys but were not part of the automated event-based triggering. For both time-based and event-based surveys, participants were instructed to complete the survey within 15 minutes of the prompt. They were reminded 3 times during the 15-minute validity period of the questionnaire: after 5 minutes, after 10 minutes, and 3 minutes before the questionnaire expired. The survey was sent to the server when it was completed or after the time for completion had expired (even when not completed).

Consumed meals, snacks, and drinks (except for water) were monitored through self-initiated surveys completed at the time of consumption or shortly thereafter. Reminders for the completion of the self-initiated surveys were incorporated within the time-based surveys. Depending on the number of triggered event-based surveys, the maximum number of surveys a participant received per day was 17.

#### 24-Hour Dietary Recall and Food Frequency Questionnaire

The self-initiated meal, snack, and drink surveys were complemented by 3 nonconsecutive 24-hour dietary recalls (24HDR) completed either on the SACANA (Self-Administered Children, Adolescent and Adults Nutrition Assessment 24-hour Dietary Recall in Czech Republic, Germany and Ireland) or the NutriNet-Santé [35] online platforms. Both instruments are validated web-based programs for the assessment of 24HDR [36-38]. Participants were asked to complete at least three 24HDR on 2 nonconsecutive weekdays and 1 weekend day during the 9 days of data collection. They were encouraged to complete the first 24HDR after the devices had been worn for at least 2 days. The online platforms provided a timestamp for each eating, snacking, or drinking occasion, place of consumption, company, and simultaneous activities. Thus, the 24HDR tools also provided data for cross-linking the context of snacking or eating behavior with the EMA data. Participants were further asked to report the frequency of intake of food items during the previous 4 weeks using a food frequency questionnaire. The food frequency questions were grouped into 15 food groups, namely vegetables, fresh fruits, drinks, breakfast cereals, milk, yogurt, fish, meat and meat products, eggs and mayonnaise, meat replacement products and plant-based products, cheese, spreadable products, olive oil, cereal products, and snacks [39].

Since measurement error is inevitable, correction methods are strongly advised to reduce bias in effect estimates when analyzing 24HDR data [40]. Therefore, usual dietary intake will be calculated according to the National Cancer Institute method [41,42] using the 24HDR and food frequency questionnaire data from the general questionnaire. Upon the completion of the study, participants completed a feasibility and acceptability questionnaire designed to assess study procedures used within the laboratory and during the 9-day protocol period.

### Data Storage and Management

All data comprising questionnaires and device data were compiled, processed, and stored in a quality-controlled common database in accordance with European Union data protection regulations. The accuracy of the EMA reports was evaluated using the activPAL’s CREA classification, which served as a gold standard for scarcely labeled free-living data [43]. The activPAL device was selected due to its unobtrusive and strategic placement on the body, enabling reliable continuous monitoring over 24-hour periods [44]. Data completeness was verified on a daily basis for each sensor to ensure integrity, and potential nonwear time was identified through 2 complementary approaches: participant self-reports collected via questionnaires and automated predictions derived from either the activPAL’s CREA algorithm or the GGIR package. This approach provided a systematic framework to assess data loss, ensure data quality, and mitigate limitations associated with the accuracy of self-reported labels. Fitbit device data and EMA prompted via the HealthReact software are stored on a European Union server with project-specific access. Fitbit data were initially transmitted and stored on a US server before retrieval by the HealthReact app. In accordance with the WEALTH standard operating procedures specifications and to ensure data quality management measures were upheld, daily monitoring processes were implemented to evaluate participant adherence or nonadherence across all centers. All study sites received email notifications regarding data inconsistencies, and the subsequent resolution of queries was conducted to increase data completeness and data quality. Identified discrepancies included inconsistent data, absent data (relevant EMAs, context-based questionnaire), range validations, and deviations from the protocol. In addition, weekly rollouts for monitoring the compliance with 24HDR entries and the general questionnaire were provided to all centers.

### Data Analysis Plan

The data collected in the WEALTH project will be used initially for 2 purposes. For hip- and wrist-worn devices, ML models will be developed to classify PB and EB from raw tri-axial accelerometer data. Model development will be based on strict labeled data from the semistructured protocol as well as labeled data from EMA time stamps with valid behavior reports of participants during the free-living survey. Classifiers for PBs and EB will be modeled using multiple ML methods ranging from random forests to convolutional neural networks or more complex deep learning applications. In the German subsample, the data on energy expenditure from the semistructured protocol will be used to develop ML models estimating metabolic equivalent of task units to each behavior.

Predictive models will be developed using the lab-based protocol and subsequently externally validated and calibrated using free-living sensor data labeled via EMA responses. To ensure robust evaluation, a unified validation strategy will be applied, combining subject-independent holdout validation, k-fold cross-validation, and performance testing on EMA-labeled free-living data. Model performance will be assessed using established metrics, including balanced accuracy, precision, recall, and *F*-score, computed only on data not used for training.

To ensure comparability across devices, accelerometer data will be harmonized by resampling to 20 Hz, converting to gravitational units (g), and synchronizing all sensors using the start time recorded during the laboratory session. EMA, accelerometer, and dietary recall data will be aligned using time stamps, enabling consistent temporal integration of self-reported and sensor-derived behaviors.

EMA-labeled data used to train ML models will be further validated by comparing them with external references, including the activPAL CREA algorithm and established ActiGraph classification methods. Low-agreement segments will be excluded using threshold-based criteria, ensuring that only high-confidence labels contribute to model development as done in Sigcha et al [43]. Model success will also be assessed through consistency across devices and quantitative agreement between EMA reports, accelerometer-derived predictions, and dietary-recall events. Agreement will be evaluated using temporal overlap metrics, Bland-Altman comparisons, intraclass correlation coefficients, and concordance indices, such as Cohen’s or weighted kappa for categorical outcomes.

The second purpose of the WEALTH study is to evaluate the potential of the use of EMA methods to study the interrelation between PB and EB. Interrelationships will be examined between PB and EB as predicted from ML models and data on context as assessed by EMA time- and context-sensitive results on incident behaviors. Multibehavioral patterns on the data from these different sources will be identified to investigate the association on body composition, well-being, and health status (self-rated health and health-related quality of life). Based on the feedback questionnaires, feasibility will be evaluated for the included measurement methods, and factors (eg, country, age, gender, activity level) associated with the degree of acceptance will be defined.

### Power Analysis and Sample Size

Sample size in each test center was guided by an estimate for a sample needed to develop the ML models. The sample size required to train a ML model successfully depends on the complexity of the model, the use of prior knowledge within the model, and the acceptable difference between performance observed during training and performance that can be expected on yet unseen data. It was estimated that a sample of at least 100 participants would allow for a substantial range of ML models to be trained successfully. This estimate is further supported by evidence from large-scale free-living accelerometer datasets, where empirical learning-curve analyses demonstrate that model performance improves markedly with increasing sample size up to approximately 60‐100 participants, after which performance gains plateau, with only marginal improvements (<1%‐2% in macro-F1) for both classical ML algorithms (eg, random forest, gradient boost), and deep learning algorithms (eg, convolutional or recurrent neural networks) [45]. These findings suggest that a cohort of 100 participants provides sufficient data to train robust, population-level models while maintaining generalizability across diverse activity contexts. To ensure that the amount of available data would not be a limiting factor in the choice of ML models, and to safeguard against data loss or participant attrition, 150 participants were recruited in each center.

### Ethical Considerations

This multicenter study is being conducted in accordance with globally accepted standards of good research practice in agreement with the Declaration of Helsinki, and with local institutional research policies, procedures, and regulations. The study design was developed by academic and clinical expertise underpinned by scientific evidence that emphasizes the requirement for standardized measurement of PBs and EBs. Patients or the public were not involved in the development of the study protocol.

Ethics committee approval was granted in each of the 4 study centers prior to study commencement. In the University of Limerick, ethics committee approval was granted by the Education and Health Sciences Faculty Research Ethics Committee (approval no 22_09_10_EHS_); in Bremen, approval was granted by the Ethics Committee of the University of Bremen (approval no 2022‐25); in the Czech Republic, approval was granted by the Committee for Research Ethics at the University of Hradec Kralove (approval no 11/2022); and in France, approval was by Comité de Protection des Personnes CPP Ile-de-France VI (approval no 2022-A02208-35).

All participants received detailed information about the purpose, procedures, risks, and benefits of the study prior to participation. Written informed consent was obtained from all participants before data collection commenced. Participants were informed of their right to withdraw at any time without penalty. Participants’ privacy and confidentiality were strictly protected. Identifying details (including names, initials, , or other personal identifiers) have been omitted.

JPI-WEALTH partners abided by the guidelines on consent [46] and transparency [47] as adopted by the European Data Protection Board and will remain in line with any new guidance that may be issued by the European Data Protection Board. The JPI-WEALTH project partners conform to the relevant convention and protocols of the Council of Europe, including the Convention on Human Rights and Biomedicine [48], and protocols on Human Rights and Biomedicine [49]. In addition, data collection was in accordance with the International Epidemiological Association’s Guidelines for Proper Conduct of Epidemiological Research [50] and the Council for International Organizations of Medical Science’s International Ethical Guidelines for Health-related Research Involving Humans [51].

## Results

The WEALTH Project was funded by the Joint Programming Initiative a Healthy Diet for a Healthy Life, a research and innovation initiative of EU member states and associated countries under grant agreement number 727565, under STAMIFY. The data collection in 2023 to 2024 resulted in rich datasets including 627 participants providing data from 2 research-grade and 2 consumer-grade devices, lab-based activity measurement, a 9-day free-living data collection period which combined the assessment of PBs and EBs via wearable devices and time-based, event-based, and self-initiated EMA, complemented by three 24HDRs. The final sample includes 627 participants, of which 44% (n=275) were male. The mean age was 32.7 (SD 13.3) years, and the mean body mass index was 24.5 (SD 4.0) kg/m². Data processing and machine-learning model development are currently underway, with primary results expected to be published in 2026.

## Discussion

### Principal Findings

The WEALTH project is expected to generate one of the most comprehensive multicountry datasets integrating accelerometer-derived PBs, wearable-derived EBs, and EMA data collected in both controlled and free-living settings. Based on the study design, we anticipate producing (1) harmonized, labeled datasets for PB and EB classification, (2) ML models capable of classifying behavioral states from raw accelerometry, and (3) an evidence-based methodology to support the simultaneous measurement of PBs and EBs in real-world contexts. These data will support the development of harmonized, labeled datasets and ML models capable of classifying PBs and EBs from raw accelerometry. This is essential given longstanding concerns about the lack of standardized device protocols, heterogeneity in cut points, and poor comparability across studies and countries [5,6,45,46]. The outputs from WEALTH will be accessible through a dedicated website outlining the project’s aims, methods, and products [52]. Critically, the ML models developed will be publicly available, enabling researchers to apply them to similar data. Providing openly accessible models and processing frameworks will facilitate detailed monitoring and assessment of PBs, EBs, and their determinants, thereby supporting future research beyond the project’s duration and contributing to data sharing and protocol harmonization.

The wearable dataset collected in this study will be made publicly available following the completion of the study and the end of an embargo period, in line with data protection and ethical approvals. The data will be shared following the Findable, Accessible, Interoperable, and Reusable (FAIR)principles, starting with a comprehensive data catalog and detailed data description, to facilitate transparency, reproducibility, and further research in this domain. During the embargo period, these data are available upon reasonable request in agreement with the research team.

Prior research has demonstrated the potential of ML to improve the classification of PB beyond traditional cut-point methods [11,12,45]. However, most existing work has been constrained to single-device datasets, laboratory environments, or narrow demographic groups. Similarly, while emerging studies have shown that wrist-worn sensors can help detect eating episodes and components of meal behaviors [15-18], free-living detection remains challenging due to variability in movement patterns and context.

Existing EMA studies have demonstrated strong value in capturing contextual, emotional, and environmental correlates of behavior [4,19] but have rarely integrated automated event-triggered surveys based on wearable sensor data. Previous work has also been limited by participants needing to self-initiate logs, contributing to recall bias and incomplete data [4]. The WEALTH design advances this field by combining 4 wearable devices, automated EMA triggers, multicountry standardization, 24HDR data, and cross-device timestamp harmonization. To our knowledge, this integrated approach has not previously been attempted at this scale in free-living behavioral research. This level of methodological integration directly addresses persistent methodological challenges documented in the literature, including cross-device comparability and harmonization of accelerometry outputs [5,6], the limited contextual fidelity of self-report and EMA-based behavioral monitoring [4,19], and the substantial complexities associated with accurately detecting and classifying EBs using wearable sensing technologies in free-living settings [15-18].

The aspiration is for WEALTH methodologies to be implemented in national and international nutritional and PA surveillance efforts. The ultimate goal is to establish a more robust system for assessing real-world data on PBs, EBs, and their determinants. This enhanced assessment capability will aid in designing public health interventions that promote health-enhancing PA and healthy diets, particularly in at-risk populations. These methodological advancements are expected to build a stronger evidence base on the health impacts of PBs and EBs, informing the development of improved national and European guidelines and recommendations aimed at enhancing citizen health. Furthermore, this accessibility is intended to support national and international efforts to achieve the United Nations Sustainable Development Goals by informing public health interventions and policies targeting PA and healthy diets in at-risk populations.

The project will deliver several key products and methodologies:

- The WEALTH taxonomy: a comprehensive classification of daily PB and EB.
- The WEALTH protocols: standardized measurement methods and integration protocols for PBs and EBs, adaptable for use in at least 4 different countries and languages.
- The WEALTH web-based infrastructure: tools for the instant processing, aggregation, and analysis of accelerometer data to classify PBs and EBs.
- The WEALTH ML models: open-access models for data processing, available via the WEALTH toolbox on the WEALTH website.
- The WEALTH detailed reports: standardized and interpretable data translation from multiple devices, facilitated by the WEALTH toolbox.
- Recommendations: guidelines for using WEALTH methods in large-scale epidemiological studies, mass surveillance, and the evaluation of intervention studies.
- Training and capacity building resources: resources for using the WEALTH toolbox, including a workshop at the final symposium and support through online teaching methodologies.

These outputs are designed to maximize the use of new knowledge and methods developed within WEALTH by project stakeholders and European Union citizens, ultimately contributing to public health improvement and policymaking. By ensuring both ML models and datasets are publicly available, WEALTH actively promotes reproducibility, transparency, and wider scientific collaboration.

### Strengths and Limitations

We used a standardized protocol across multiple countries to capture both lab-based and free-living PBs and EBs. The combination of research-grade and consumer-grade wearable devices enabled the comprehensive monitoring of participant activities and physiological responses. The 9-day EMA and 24HDR provided rich, real-time data for examining behavioral patterns in real-life contexts. The backend engineering protocols of HealthReact are proprietary due to commercial licensing, which may limit direct replicability. The intensive data collection and device reliance may have introduced participant burden and technological challenges, affecting data quality. Self-reported EMA and dietary data could be subject to recall and social desirability bias, despite correction efforts.

### Conclusion

This protocol outlines the design and methodology of the WEALTH project, a multicountry initiative to develop standardized, integrated approaches for assessing PBs and EBs using wearable sensors, ecological momentary assessment, and ML techniques. Although the findings are forthcoming, the anticipated outputs include harmonized datasets, openly accessible ML models, and an integrated data processing framework. These outputs have the potential to strengthen behavioral surveillance, support epidemiological and interventional research, and inform public health guidance. By combining rigorous standardization with advanced sensing and analytical methods, WEALTH is positioned to make a substantive contribution to the harmonization of PB and EB measurement across Europe and to future digital health innovation.

## Abbreviations

24HDR: 24-hour dietary recall
EB: eating behavior
EMA: ecological momentary assessment
ML: machine learning
PA: physical activity
PB: physical behavior
SACANA: Self-Administered Children, Adolescent and Adults Nutrition Assessment
STAMIFY: standardised measurement, monitoring and/or biomarkers to study food intake, physical activity and health
WEALTH: Wearable Sensor Assessment of Physical and Eating Behaviours
